# A Novel Approach to Raman Distributed Temperature-Sensing System for Short-Range Applications

**DOI:** 10.3390/s24092669

**Published:** 2024-04-23

**Authors:** Augusto Pieracci, Jacopo Nanni, Giovanni Tartarini, Massimo Lanzoni

**Affiliations:** Department of Electrical, Electronic and Information Engineering “Guglielmo Marconi”, University of Bologna, 40136 Bologna, Italy; augusto.pieracci@unibo.it (A.P.); giovanni.tartarini@unibo.it (G.T.); massimo.lanzoni@unibo.it (M.L.)

**Keywords:** Raman scattering, distributed temperature sensors, optical fiber sensors

## Abstract

A novel approach to the development of Distributed Temperature-Sensing (DTS) systems based on Raman Scattering in Multimode optical fibers operating at around 800 nm is presented, focusing on applications requiring temperature profile measurement in the range of a few hundreds of meters. In contrast to the standard Raman DTS systems, which aim to shorten the pulse space width as much as possible to improve the precision of measurement, the novel approach studied in this work is based on the use of pulses with a space width that is approximately equal to the distance covered by the fiber under test. The proposed technique relies on numerical post-processing to obtain the temperature profile measurement with a precision of about ±3 °C and a spatial resolution of 8 m, due to the transaction phases of the optical pulses. This solution simplifies the electronic circuit development, also minimizing the required laser peak power needed compared to the typical narrow pulse techniques.

## 1. Introduction

Distributed Temperature Sensors (DTS) realized through optical fibers, which exploit the nonlinear interactions of the Brillouin and Raman types, have been an active area of research for numerous years, and now constitute the physical core of a great variety of commercial products tailored for different customer needs. Indeed, they exploit the intrinsic advantages of optical fibers, namely low signal attenuation, immunity to electromagnetic interference, and reduced dimensions, in combination with their ability to provide fully distributed temperature measurements [1,2].

In the Brillouin case, the physical phenomenon on which the sensing is based is the temperature dependence of the Brillouin frequency shift, which takes place at each section of the fiber once it is hit by a given optical pump signal. For this purpose, the Brillouin Optical Time Domain Reflectometry (BOTDR) technique exploits a pulsed pump laser and, in order to detect correctly the backscattered signal, performs its detection within a coherent optical scheme where a relatively strong power of the local oscillator is required [3]. The Brillouin Optical Time Domain Analysis (BOTDA) is instead based on the interaction of two counterpropagating waves, one of which is pulsed while the other is a continuous wave (CW), and requires access at both ends of the fiber under test (FUT) [4]. Because of the typical stronger levels of the detected signal, BOTDA features the capability to be successfully applied to FUTs with lengths of the order of kilometers/tens of kilometers, with spatial resolutions of the order of centimeters/tens of centimeters [5,6,7].

As regards Raman-based DTS, the basic physical phenomenon exploited is the temperature dependence of the Anti-Stokes Raman Scattering Coefficient [8]. The technique of Optical Frequency Domain Reflectometry (OFDR) can be adopted in this context, where an optical signal, whose frequency is linearly swept in time, is sent into the FUT, and the temperature of a given section is obtained through a measurement of the time-varying beating frequency within a self-homodyne optical detection scheme [9]. The OFDR technique, which can also be utilized with the same purpose in combination with the linear Rayleigh backscattered signal [10,11], indeed allows a temperature analysis with a very high spatial resolution between the different sections of the FUT. At the same time, the total length that can be analyzed depends on the coherence length of the optical source utilized, which should consequently be of superior high quality, if FUTs longer than a few tens of meters have to be analyzed. One possibility to increase the cost effectiveness of OFDR is the so-called Incoherent OFDR (I-OFDR) technique, in which the sweeping is not performed on the optical frequency, but on the envelope of the optical source [12]. Raman temperature sensors based on the I-OFDR technique [13] have been indeed successfully proposed that allow consideration of FUTs of relatively higher lengths [14].

The exploitation of the temperature dependence of the Raman Anti-Stokes backscattered signal can also be performed through the Optical Time Domain Reflectometry (R-OTDR) technique [15,16]. In this case, the system to be realized is rather simple, since the measurement is performed by launching into the optical fiber a periodical sequence of optical pulses, and monitoring the Raman components of the backscattered light with the aid of an optical coupler/optical circulator followed by an appropriate optical filter [17]. This aspect, in conjunction with the high effectiveness of the sensing operation performed, led to the widespread utilization of R-OTDR DTS, which includes the areas of electrical power distribution [18], the oil and gas industry [19], hydrologic systems [20], and geology [21]. In general, for these applications the lengths of the temperature-monitoring fibers can easily reach a few km to a few tens of km. Given these values, to guarantee appropriate levels of the required figures of merit, like spatial resolution, temperature resolution, and reliability of the temperature value, high-quality commercial R-OTDR-DTS-based systems can be found in the market, and the related expenditure can be regarded as acceptable for the concerned public body or private company.

A particularly relevant application which falls into the area of geology is that of monitoring the temperature at different depths of boreholes to be utilized for heat exchanging. This use of R-OTDR-DTS is employed in the context of ground-source heat pump (GSHP) systems, which are used for space heating and cooling, and offer an energy-efficient alternative to traditional systems based on fossil fuel [22]. In this framework, the borehole temperature monitoring can be performed through the adoption of commercially available R-OTDR-DTS systems [23]. However, in the case where residential houses or small offices are considered, this additional purchase can constitute an important effort for the final user. It is therefore desirable to have at disposal a R-OTDR-DTS system tailored for these scenarios, which guarantees the required monitoring while also maintaining the global cost of the system at acceptable levels.

In this paper, a preliminary cost-efficient R-OTDR-DTS realization to be utilized in the GSHP context is presented. The system has been chosen to operate in the first optical window. Indeed, high-power lasers are less expensive in this case because they can be more easily realized with respect to the third optical window, and, at the same time, the higher value exhibited by the fiber attenuation at these short wavelengths does not constitute a problem given the low spans (very few hundreds of meters) of sensing fiber considered. Moreover, a novel approach for signal post-processing is adopted, which relaxes the value of the maximum instantaneous power required to the pulse generator and allows the use of off-the-shelf electronic components, obtaining a R-OTDR-DTS system with a global high performance-to-cost ratio.

In the following, some theoretical basic concepts on which the system is based are presented. Subsequently, the experimental setup is described, and the measurement results are reported. The proposed post-processing of such results is then illustrated, showing the capability of the system to act as a low-cost reliable R-OTDR-DTS for short-range applications. Finally, conclusions are drawn.

## 2. Mathematical Model and Proposed Approach

**Figure 1:**
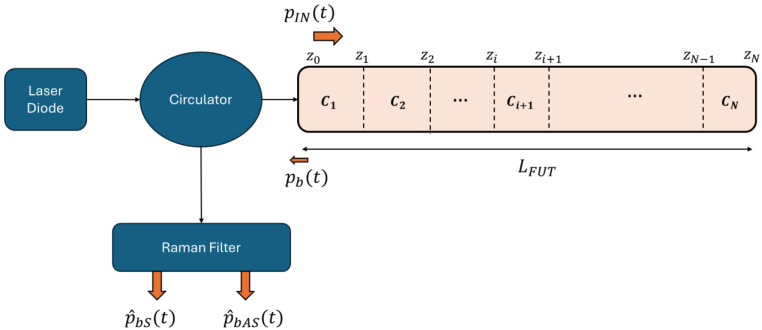
Scheme utilized to develop the theoretical model of the backscattering process.

Starting from the scheme shown in Figure 1, an optical pulse generated by a high-power laser diode (HPLD) operating at $\lambda_{0}=808\;nm$ is considered entering a circulator, which forwards the pulse $p_{IN}\left(t\right)$ to the optical fiber under test (FUT). Within the fiber, *N* separated sections are considered, over which the field undergoes the backscattering process with a related coefficient $C_{i}$ related to the i-th section of longitudinal coordinate z, generating the overall backscattered field $p_{b}\left(t\right)$, which contains the desired information about the temperature. Then, the field $p_{b}\left(t\right)$ enters the circulator, which forwards the signal to the Raman filter, designed to separate the Stokes and Anti-Stokes components into two paths, by filtering as much as possible the Rayleigh and Brillouin components.

The total amount of received power at the fiber-input section, due to backscattering and reflections, will include Stokes and Anti-Stokes Raman components, having center frequency values around $\nu_{0}-\Delta \nu =\frac{c}{\lambda_{S}}$ and $\nu_{0}+\Delta \nu =\frac{c}{\lambda_{AS}}$ with Δν≃12 THz [24], where c is the speed of light, while $\lambda_{S}$ and $\lambda_{AS}$ are the central wavelengths of Stokes and Anti-Stokes Raman components, respectively. Moreover, the received power will also comprise Rayleigh and Brillouin backscattering, and Fresnel’s reflections components, all centered for simplicity at $\nu_{0}=\frac{c}{\lambda_{0}}$, considering the high proximity of Brillouin compared to Rayleigh and Fresnel components (i.e., separation of 11 GHz).

The expression of the generic infinitesimal power, which is reflected by an infinitesimal section, can be written as follows:

(1) $$dp_{bAS}\left(t,z=0\right)=C_{AS}\left(T(z)\right)\;p_{IN}\left(t-\frac{2z}{v_{g}(\lambda_{AS})}\right)e^{-2\alpha \left(\lambda_{AS}\right)\;z}\;dz$$

(2) $${dp}_{bS}\left(t,z=0\right)=C_{S}\left(T(z)\right)\;p_{IN}\left(t-\frac{2z}{v_{g}(\lambda_{S})}\right)e^{-2\alpha \left(\lambda_{S}\right)z}\;dz$$

(3) $$dp_{bR/B/F}\left(t,z=0\right)=C_{R/B/F}\left(z\right)\;p_{IN}\left(t-\frac{2z}{v_{g}\left(\lambda_{0}\right)}\right)e^{-2\alpha \left(\lambda_{0}\right)z}dz$$

where $dp_{bAS},dp_{bS},and\;dp_{bR/B/F}$ represent the portion of the backscattered field due to Raman Anti-Stokes, Raman Stokes, and Rayleigh/Brillouin/Fresnel, respectively, where the Fresnel coefficients are indeed related only for the first and last connection (i.e., z=0 and $z=L_{FUT}$).

Moreover, $\alpha \left(\lambda \right)$ is the power attenuation constant at the specific wavelength considered and $v_{g}(\lambda )$ is the group velocity in the fiber, where both quantities are wavelength dependent.

Finally, the expressions of the Anti-Stokes and Stokes coefficients, named $C_{AS}\left(T(z)\right)$ and $C_{S}\left(T(z)\right)$, respectively, are given by [24]:

(4) $$C_{AS}\left(T(z)\right)=\Gamma_{AS}\frac{e^{-\frac{h\Delta \nu }{kT(z)}}}{1-e^{-\frac{h\Delta \nu }{kT(z)}}}$$

(5) $$C_{S}\left(T(z)\right)=\Gamma_{S}\frac{1}{1-e^{-\frac{h\Delta \nu }{kT(z)}}}$$

where $\Gamma_{AS},\;\Gamma_{S}$ are the related capture coefficients, T(z) is the value of the temperature at the coordinate z, and h,k are the Planck’s and Boltzmann’s constants, respectively.

The total backscattered power $p_{b}\left(t\right)$ at the section z=0 is then given by the sum of the different scattering contributions as shown in the following expression:

(6) $$p_{b}\left(t\right)=\int_{0}^{L_{FUT}}dp_{bAS}+\int_{0}^{L_{FUT}}dp_{bS}+\int_{0}^{L_{FUT}}dp_{bR/B/F}=p_{bAS}\left(t\right)+p_{bS}\left(t\right)+p_{bR/B/F}\left(t\right)$$

where $dp_{bAS},dp_{bS},dp_{bR/B/F}$ are given by (1)–(3).

Exploiting the Fourier analysis, it is now straightforward to perform numerical simulations and extrapolate the output signals from the Raman filter, taking also into account its response. By applying the Fourier analysis, it is possible to write:

(7) $$p_{bAS}\left(t\right)=F^{-1}\left\{P_{bAS}\left(\omega \right)\right\}=F^{-1}\left\{\int_{0}^{L_{FUT}}C_{AS}\left(T(z)\right)P_{IN}\left(\omega \right)e^{-j\omega \frac{2z}{v_{g}\left(\lambda_{AS}\right)}}\;e^{-2\alpha \left(\lambda_{AS}\right)z}dz\right\}$$

(8) $$p_{bS}\left(t\right)=F^{-1}\left\{P_{bS}\left(\omega \right)\right\}=F^{-1}\left\{\int_{0}^{L_{FUT}}C_{S}\left(T(z)\right)P_{IN}\left(\omega \right)e^{-j\omega \frac{2z}{v_{g}\left(\lambda_{S}\right)}}\;e^{-2\alpha \left(\lambda_{S}\right)z}dz\right\}$$

(9) $$p_{bR/B/F}\left(t\right)=F^{-1}\left\{P_{bR/B/F}\left(\omega \right)\right\}=C_{R/B/F}F^{-1}\left\{\int_{0}^{L_{FUT}}P_{IN}\left(\omega \right)e^{-j\omega \frac{2z}{v_{g}\left(\lambda_{0}\right)}}\;e^{-2\alpha (\lambda_{0})z}dz\right\}$$

where $P_{bAS}\left(\omega \right),\;P_{bS}\left(\omega \right)$, and $P_{bR/B/F}\left(\omega \right)$ are the Fourier Transforms of $p_{bAS}\left(t\right),\;{\;p}_{bS}\left(t\right)$, and $p_{bR/B/F}\left(t\right)$, respectively, $P_{IN}\left(\omega \right)$ is the Fourier Transform of the input fiber signal $p_{IN}\left(t\right)$, and the operator $F^{-1}\{\cdot \}$ represents the Inverse Fourier Transform.

The Raman filter can then be modeled as an optical duplexer that separates the Raman Stokes and Anti-Stokes frequencies. In the ideal case, the Rayleigh/Brillouin/Fresnel backscattered field would be filtered out, but in practice such a contribution will just undergo an attenuation, which will be quantified through the factors $A_{R/B/F,AS},A_{R/B/F,S}$ for Anti-Stokes and Stokes ports, respectively.

Therefore, the final expression of the two output powers from the Raman filter are:

(10) $${\hat{p}}_{bAS}\;\left(t\right)=F^{-1}\left\{\int_{0}^{L_{FUT}}C_{AS}\left(T(z)\right)P_{IN}\left(\omega \right)e^{-j\omega \frac{2z}{v_{g}\left(\lambda_{AS}\right)}}\;e^{-2\alpha \left(\lambda_{AS}\right)z}dz\right\}+\;A_{R/B/F,\;AS}\;C_{R/B/F}F^{-1}\left\{\int_{0}^{L_{FUT}}P_{IN}\left(\omega \right)e^{-j\omega \frac{2z}{v_{g}\left(\lambda_{0}\right)}}\;e^{-2\alpha (\lambda_{0})z}dz\right\}$$

(11) $${\hat{p}}_{bS}\;\left(t\right)=F^{-1}\left\{\int_{0}^{L_{FUT}}C_{S}\left(T(z)\right)P_{IN}\left(\omega \right)e^{-j\omega \frac{2z}{v_{g}\left(\lambda_{S}\right)}}\;e^{-2\alpha \left(\lambda_{S}\right)z}dz\right\}+A_{R/B/F,\;S}\;C_{R/B/F}F^{-1}\left\{\int_{0}^{L_{FUT}}P_{IN}\left(\omega \right)e^{-j\omega \frac{2z}{v_{g}\left(\lambda_{0}\right)}}\;e^{-2\alpha (\lambda_{0})z}dz\right\}$$

These integrals can then be evaluated section by section assuming that the coefficients $C_{AS},C_{S}\;$ are constant in the *i*-th section, namely $C_{i,AS},C_{i,S}$, obtaining the following expressions:

(12) $${\hat{p}}_{bAS}\;\left(t\right)=F^{-1}\left\{P_{IN}\left(\omega \right)\sum_{i=1}^{N}C_{i,AS}\int_{z_{i-1}}^{z_{i}}e^{-j\omega \frac{2z}{v_{g}\left(\lambda_{AS}\right)}}\;e^{-2\alpha \left(\lambda_{AS}\right)z}dz\right\}+A_{R/B/F,\;AS}\;C_{R/B/F}F^{-1}\left\{P_{IN}\left(\omega \right)\int_{0}^{L_{FUT}}e^{-j\omega \frac{2z}{v_{g}\left(\lambda_{0}\right)}}\;e^{-2\alpha \left(\lambda_{0}\right)z}dz\right\}$$

(13) $${\hat{p}}_{bS}\;\left(t\right)=F^{-1}\left\{P_{IN}\left(\omega \right)\sum_{i=1}^{N}C_{i,S}\int_{z_{i-1}}^{z_{i}}e^{-j\omega \frac{2z}{v_{g}\left(\lambda_{S}\right)}}\;e^{-2\alpha \left(\lambda_{S}\right)z}dz\right\}+A_{R/B/F,S}\;C_{R/B/F}F^{-1}\left\{P_{IN}\left(\omega \right)\int_{0}^{L_{FUT}}e^{-j\omega \frac{2z}{v_{g}\left(\lambda_{0}\right)}}\;e^{-2\alpha \left(\lambda_{0}\right)z}dz\right\}$$

Note that because a very short distance (of the order of hundreds of meters) is considered in this particular application, both intermodal and chromatic dispersion effects can be neglected. A dependence on the temperature T(z) is expected both in ${\hat{p}}_{bAS}\;\left(t\right)$ and ${\hat{p}}_{bS}\;\left(t\right)$ which, due to (4) and (5), is expected to be relatively strong in the former and relatively weak in the latter.

As mentioned in the Introduction, the objective of the present work is to illustrate an approach which allows the realization of a cost-effective R-DTS to be applied to distances of a few hundred meters. In particular, the proposed system does not require driving the laser with ultra-short current pulses with very high instantaneous power.

The consideration on which this approach is based is that, instead of modulating the laser with short current pulses and monitoring the behaviors of ${\hat{p}}_{bAS}\;\left(t_{j}\right)$ and ${\hat{p}}_{bS}\;\left(t_{j}\right)$, where $t_{j}=\frac{{2z}_{j}}{v_{g}},\;j=0,\ldots ,N$ is the instant at which the contribution of reflected power coming from section $z=z_{j}$ reaches the input section of the fiber, it is possible to extract the information on the value of the temperature at the different sections $z_{j}$ of the fiber utilizing long current pulses and monitoring the behaviors of the differences $\delta {\hat{p}}_{bAS}\;\left(t_{j}\right)={\hat{p}}_{bAS}\;\left(t_{j}\right)-{\hat{p}}_{bAS}\;\left(t_{j-1}\right)$ and $\delta {\hat{p}}_{bS}\;\left(t_{j}\right)={\hat{p}}_{bS}\;\left(t_{j}\right)-{\hat{p}}_{bS}\;\left(t_{j-1}\right)$.

An intuitive demonstration of the correctness of this approach can be given considering ${\hat{p}}_{bAS}\;\left(t\right)$ or ${\hat{p}}_{bAS}\;\left(t\right)$ given respectively by (12) and (13), in the case when no Rayleigh/Brillouin/Fresnel contributions are present, assuming at the same time that the attenuation terms can be neglected and that the reflections are concentrated at the fiber sections of coordinate $z_{i}=i∆z.$ In such a case, starting for example from (12), it is possible to write:

(14) $${\hat{p}}_{bAS}\;\left(t\right)=F^{-1}\left\{P_{IN}\left(\omega \right)\sum_{i=1}^{N}C_{i,AS}∆ze^{-j\omega \frac{{2z}_{i}}{v_{g}}}\right\}=\sum_{i=1}^{N}C_{i,AS}∆zp_{IN}\left(t-\frac{{2z}_{i}}{v_{g}}\right)$$

Considering (14) for ${t=t}_{j}$ thinking initially of a value j<N, it can be written:

(15) $${\hat{p}}_{bAS}\;\left(t_{j}\right)=\sum_{i=1}^{j}C_{i,AS}∆zp_{IN}\left(t_{j}-\frac{{2z}_{i}}{v_{g}}\right)$$

where the contributions to ${\hat{p}}_{bAS}\;\left(t_{j}\right)$ come, on one hand, from the reflection at ${z=z}_{j}$, for which it is $p_{IN}\left(t_{j}-\frac{{2z}_{j}}{v_{g}}\right)=p_{IN}\left(0\right)$, i.e., ${z=z}_{j}$ reflects the initial portion of the pulse, and, on the other hand, from all the sections ${z=z}_{i},\;i=0,\ldots ,\;j-1$, which, due to the long duration of the pulse, reflect its delayed portions. Applying the same reasoning to ${\hat{p}}_{bAS}\;\left(t_{j-1}\right)$ and assuming the pulse to be ideally rectangular, it is a straightforward result that, from the difference ${\hat{p}}_{bAS}\;\left(t_{j}\right)-{\hat{p}}_{bAS}\;\left(t_{j-1}\right)$, the contribution due to the only *j*-th fiber section can be extracted. In this simplified example, this difference is $C_{j,AS}∆z=C_{AS}\left(T(z_{j})\right)∆z$, from which the temperature at $z=z_{j}$ can be directly extracted.

The MATLAB modeling program that has been developed, based on the illustrated theoretical model, utilizes however the rigorous expressions ${\hat{p}}_{bAS}\;\left(t\right)\;$ and ${\hat{p}}_{bS}\;\left(t\right)$, allowing numerical results to be obtained that can be utilized for a validation of the experimental ones. Such a simulation program was then utilized to qualitatively prove the effectiveness of the proposed approach.

The behaviors of $p_{IN}\left(t\right)$ coincide with the ones that have been effectively utilized in the experiments, and their descriptive parameters are summarized in Table 1.

**Figure 2:**
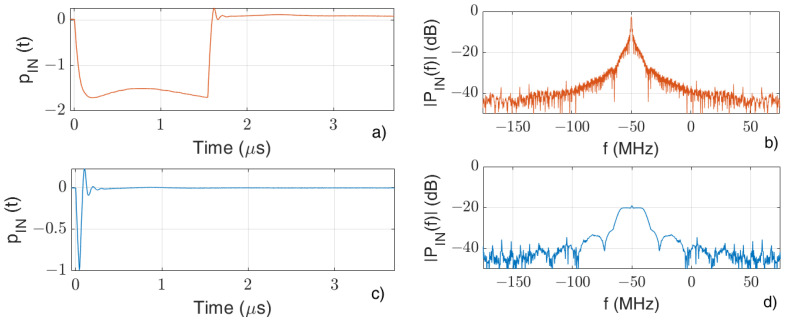
“Long pulse” time (**a**) and frequency (**b**) behaviors, and “Short pulse” time (**c**) and frequency (**d**) behaviors.

The time behaviors of $p_{IN}\left(t\right)$ considered for the simulations in the long and short pulse cases are shown in Figure 2 together with their related spectra.

The simulations were performed considering a total fiber length $L_{FUT}=z_{N}=150\;m$ and three temperature profiles:

- Test 1: All of the fiber at 25 °C;
- Test 2: First 100 m at 60 °C and the remaining 50 m at 25 °C;
- Test 3: First 100 m at 3 °C and the remaining 50 m at 25 °C.

**Figure 3:**
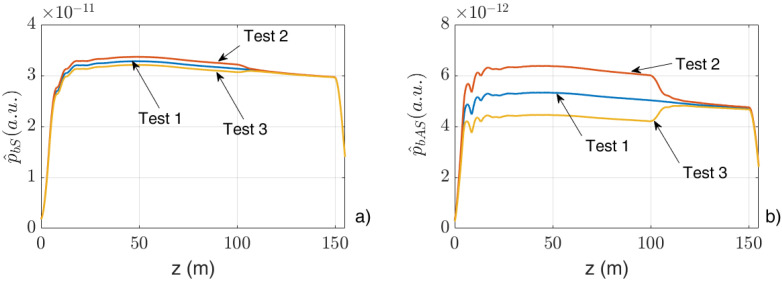

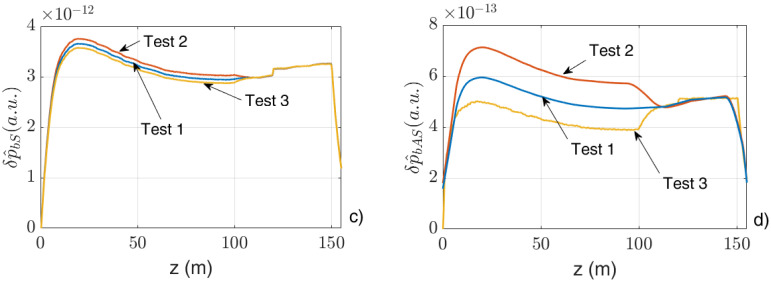
Behaviors of (**a**) ${\hat{p}}_{bS}\left(t\right)$ and (**b**) ${\hat{p}}_{bAS}\left(t\right)$ for the case of the “Short pulse” and of the differences (**c**) $\delta {\hat{p}}_{bS}\left(t\right)$ and (**d**) ${\delta \hat{p}}_{bAS}\left(t\right)$ for the case of “Long pulse”.

Figure 3 show the behaviors of ${\hat{p}}_{bS}\;$ and ${\hat{p}}_{bAS}\;$ for the case of the “Short pulse” and the variations $\delta {\hat{p}}_{bS}\;$ and ${\delta \hat{p}}_{bAS}\;$ for the case of the “Long pulse” (in which case, a value of $t_{j}-t_{j-1}=1m/v_{g}$ was chosen, in agreement with the experimental measurements).

Note that the quantity represented by the abscissa is the longitudinal coordinate z instead of the measurement time t. Indeed, the direct correspondence $z=v_{g}t$ between the two quantities allows this substitution to be performed, which has the advantage of offering a more visualizable correspondence with the considered fiber section.

The qualitative agreement between the figures at the left with the correspondent ones at the right suggests that, by using the “Long pulse” and taking the variations $\delta {\hat{p}}_{bAS}\;$ and $\delta {\hat{p}}_{bS}\;$ of the output Anti-Stokes and Stokes signals, similar performances can be achieved compared to those of the “Short pulse”. A further confirmation of this result is given by the experimental measurements described in the following.

## 3. Experimental Setup

The experimental setup realized for this work is based on an electronic excitation circuit used to drive the laser source, an optical system suitable for filtering the backscattered radiation, and an electronic section whose function is to amplify and filter the weak signal at the output of the photodiode detector.

The whole setup is controlled by a workstation and a LabVIEW VI, which is able to collect measurement data, perform post-processing (in particular compensate for the setup non-linearity), and present final results.

**Figure 4:**
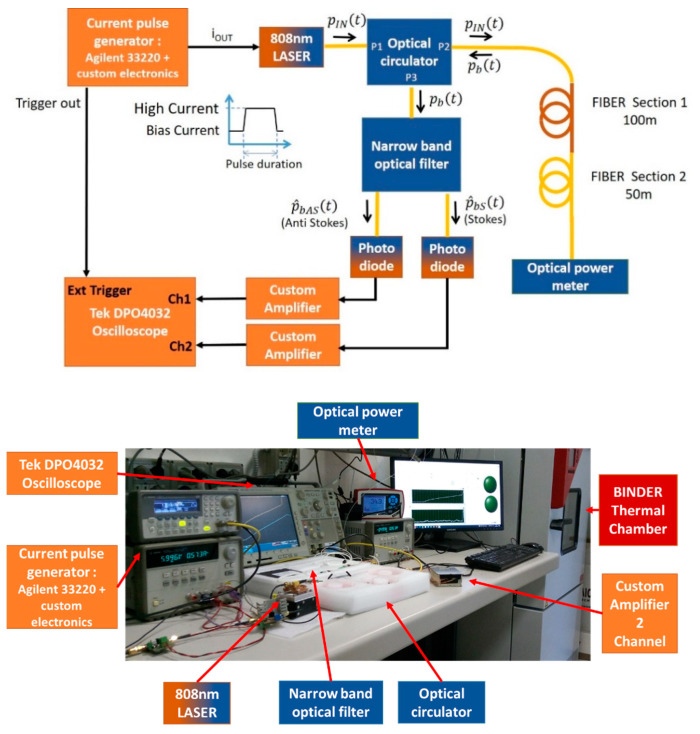
Schematic representation of the experimental setup.

A simplified schematic of the setup is shown in Figure 4.

The excitation circuit is based on a fast current pulse generator. This circuit is characterized by a very short duration of the rise and fall edges. Moreover, the low level of the current generated to drive the laser has a positive value. This is necessary in order to maintain the laser above threshold and improve the source time responsivity. The duration of the high current pulse has been varied from 80 ns (named “Short pulse”) to 1500 ns (named “Long pulse”). The laser used in this work is a MLDP-808-2W-MM from CNI LASER (Changchun, China) with a thermo-electric cooler and is able to emit up to 2 W of optical power at a wavelength of $\lambda_{0}=808\;nm$. The amplitude of the excitation current can be raised up to 1.5 A corresponding to an optical output power of 1.5 W.

As mentioned in the Introduction, the choice to operate in the first optical window is related to the fact that, on one hand, high-power lasers can be more easily realized at low wavelengths, allowing the global cost of the system to be kept at a relatively low level, and that, on the other, the higher attenuation experienced by the optical signal at these wavelengths does not introduce detriment to the system operation due to the short lengths of the fiber spans utilized.

In line with the model described in the previous section (see also Figure 1), the laser output is connected to the forward input (P1) of an optical circulator (MMCIR-808-12-M5-L-05-FA from Of-Link, Shenzhen, China), whose function is to separate the forward radiation from the one backscattered from the fiber. This latter is available at the circulator output (P3) and, as mentioned, features three main components, namely the Rayleigh/Brillouin/Fresnel term $p_{bR/B/F}\left(t\right)$, the Stokes term ${\;p}_{bS}\left(t\right)$, and the Anti-Stokes term $p_{bAS}\left(t\right)$, which are centered, respectively, at the frequencies ${c/\lambda }_{0}$, ${c/\lambda }_{0}-∆\nu$ and ${c/\lambda }_{0}+∆\nu$, with Δν≃12 THz. Note that the first term includes an important contribution coming from the Fresnel reflection, which takes place at the end of the fiber (i.e., at ${z=z}_{N}$, see Figure 1). This component reaches the circulator after a time delay equal to double the propagation time through the fiber because, once it has been reflected, it has to propagate back to the circulator along the same path. The fiber used in our experiments is MM fiber 50/125 µm of type OM2.

Still referring to Figure 4, the narrow band optical filter which is placed after the (P3) output of the circulator (780/835 nm WDM Optical filter from HAPHIT, Würzburg Germany), selects the weak Stokes and Anti-Stokes radiations ${\;p}_{bS}\left(t\right)$ and $p_{bAS}\left(t\right)$ and ideally rejects the other components. In particular, it is necessary to reject the term $p_{bR/B/F}\left(t\right)$, whose intensity, due to the mentioned term of the Fresnel reflection in ${z=z}_{N}$, is several orders of magnitude higher with respect to both ${\;p}_{bS}\left(t\right)$ and $p_{bAS}\left(t\right)$ components, and can damage (or at least temporarily blind) the high-sensitivity detector. A residual unfiltered contribution coming from $p_{bR/B/F}\left(t\right)$ is, however, unavoidable; therefore, the actual powers exiting the filter will be indicated by the terms ${\hat{p}}_{bS}\;\left(t\right)$, ${\hat{p}}_{bAS}\;\left(t\right)$, defined in (10), (11).

The optical fiber, which is connected to the (P2) output of the circulator, consists in a widely available 50 μm core Multimode fiber. Two sections of the strand, which are actually seamlessly connected, are distinguished in Figure 4, since they can be heated to different temperatures, allowing the evaluation of the effectiveness of the measurement technique with particular regard to sensitivity and spatial resolution. In particular, the optical fiber was heated to two different temperatures by means of a couple of thermostatically controlled tanks. The first section of the fiber (100 m) was heated to 3 °C, 25 °C, and 60 °C, while the second one (50 m) was maintained at 25 °C, acting as measurement check.

An optical thermopile-based power meter is placed at the end of the fiber to monitor the laser stability during the measurement, whose duration can vary from a few minutes to tens of minutes depending on the required accuracy.

Finally, the intensities of ${\hat{p}}_{bS}\;\left(t\right)$ and ${\hat{p}}_{bAS}\;\left(t\right)$ are measured by means of commonly available GaAs PIN photodiodes (GP85-FCT0N GaAs PIN from Optowell, Jeonju-si, Republic of Korea) whose responsivity at the wavelength of interest is, in both cases, 0.6 A/W. The output signal currents are converted to voltage signals and amplified by means of a low-noise two-stage amplifier based on commercial devices, obtaining as final outputs the tensions ${\hat{v}}_{bS}\;\left(t\right)$ and ${\hat{v}}_{bAS}\;\left(t\right)$, which are proportional to ${\hat{p}}_{bS}\;\left(t\right)$ and ${\hat{p}}_{bAS}\;\left(t\right)$, respectively. The total voltage gain is 12.

The weak signals at the output of the amplification chain were analyzed by means of a high-performance oscilloscope and the experimental measurements shown in this paper are the result of averaging after a suitable warmup period of the whole system.

In this regard, it is necessary to stress that the radiation of the laser source is dependent on the laser temperature, and for this reason it is equipped with a temperature controller system. However, the operating temperature is only reached after a settling time of about 30 min.

The described setup is suitable for embedded systems. The excitation and detection section are indeed based on standard electro-optical components, and data acquisition and post-processing can be easily realized by means of low-cost, compact electronics. The whole system can then be engineered in a small, lightweight, rugged, embedded setup suitable for temporary as well as permanent field installation.

## 4. Measurement, Post-Processing, and Discussion

Using the setup of Figure 4, Stokes and Anti-Stokes signals ${\hat{v}}_{bS}\;\left(t\right)$ and ${\hat{v}}_{bAS}\;\left(t\right)$ were acquired by averaging 512,000 measurements at different temperatures. The total duration of each measurement was approximately 10 min.

The measurements were performed by generating “Short pulses” and “Long pulses” with the characteristics listed in Table 1 above.

**Figure 5:**
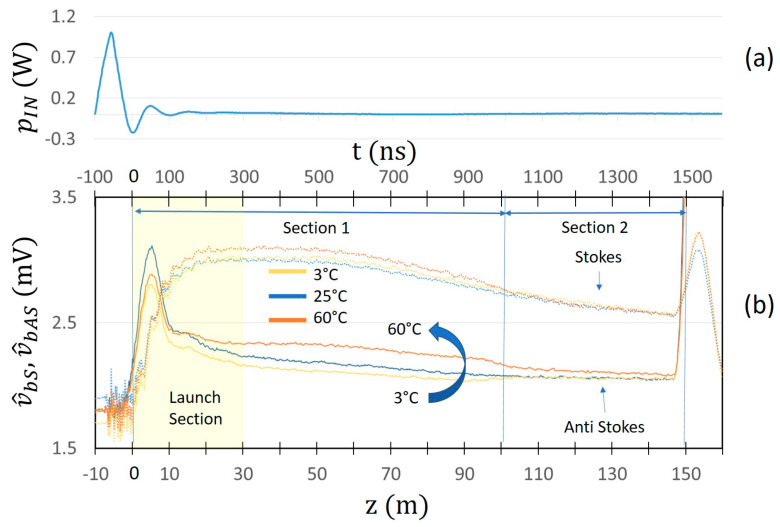
Time and longitudinal coordinate behaviors of (**a**): “Short pulse” applied to the laser (**b**): Stokes and Anti-Stokes components ${\hat{v}}_{bS}\;\left(z\right)$ and ${\hat{v}}_{bAS}\;\left(z\right)$ of the Raman backscattered signal obtained for different values of temperature of fiber Section 1.

In Figure 5a, time and longitudinal coordinate behaviors of the “Short pulse” applied to the laser are represented. The optical “Short Pulse” has a width of 82 nsec and a rise time of 50 nsec. Propagation time $t_{p}(\lambda )$ and distance traveled in the fiber $d_{t}(\lambda )$ are related by the group velocity $v_{g}(\lambda )$ through the relationship:

(16) $$d_{t}\left(\lambda \right)=t_{p}\left(\lambda \right)\;\cdot \;v_{g}(\lambda )$$

The resolution of the system is about 8 m, derived from Equation (16), where $t_{p}\left(\lambda \right)$ is the sum of the rise and falling time of the “Short Pulse” (see Table 1) and $v_{g}\left(\lambda \right)\approx 2\times 10^{8}\;m/sec$.

The correspondent averaged raw Raman signals are shown in Figure 5b. The temperature of Section 1 of the optical fiber was changed from 3 °C to 60 °C, keeping the temperature of Section 2 unchanged to 25 °C. As expected, the Anti-Stokes signal increases with temperature. As can be seen by analyzing the acquired signal, the behavior in the first 300 ns (corresponding approximately to the initial 20 m of optical fiber) is abnormal. This is a well-known effect in Optical Time Domain Reflectometry [25], and is due to the combination of Raman and Rayleigh/Fresnel back radiations caused by the non-ideality of the optical connectors. Due to this unavoidable phenomenon in the first part of the fiber (that we call the launch section), its contribution is neglected in the following discussions.

A further aspect that can be observed is a slight decrease in the response as a function of time (i.e., fiber length), even if the considered section is at constant temperature. This is due to the fiber optical attenuation and can be easily compensated for during post-processing.

**Figure 6:**
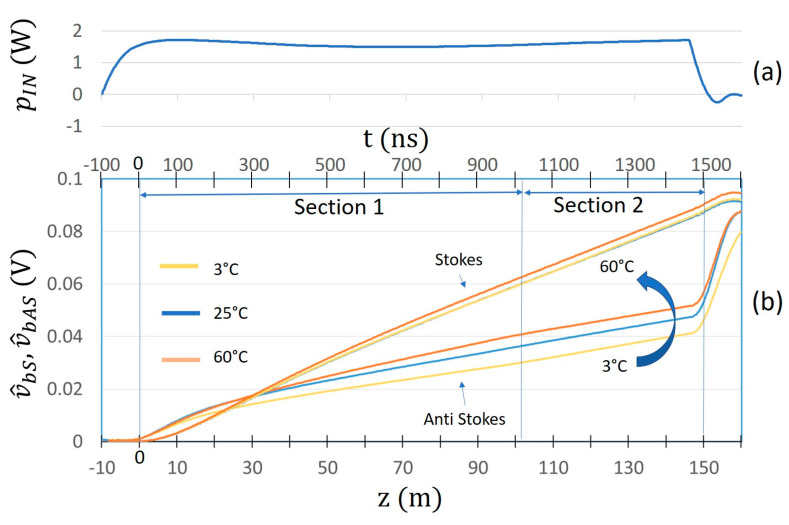
Time and longitudinal coordinate behaviors of (**a**): “Long pulse” applied to the laser (**b**): Stokes and Anti-Stokes components ${\hat{v}}_{bS}\;\left(z\right)$ and ${\hat{v}}_{bAS}\;\left(z\right)$ of the Raman backscattered signal obtained for different values of temperature of fiber Section 1.

In Figure 6a, time and longitudinal coordinate behaviors of the “Long pulse” applied to the laser are instead represented. The optical “Long Pulse” has a width of 1500 nsec and a rise time of 80 nsec. In Figure 6b, the correspondent averaged raw signal response is shown. As can be seen, the contribution of the launch fiber is increased with respect to the case of the “Short pulse”. This is due to the fact that in the case of the “Long pulse”, the rise time is much greater than in the case of the “Short pulse” for a higher peak power value.

As illustrated in Section 2, the Raman signal as a function of the fiber length can be numerically obtained by differentiating the raw signal:

(17) $${\delta \hat{v}}_{bAS}\left(z_{i}\right)={\hat{v}}_{bAS}\left(z_{i}\right)-{\delta \hat{v}}_{bAS}\left(z_{i-1}\right)$$

**Figure 7:**
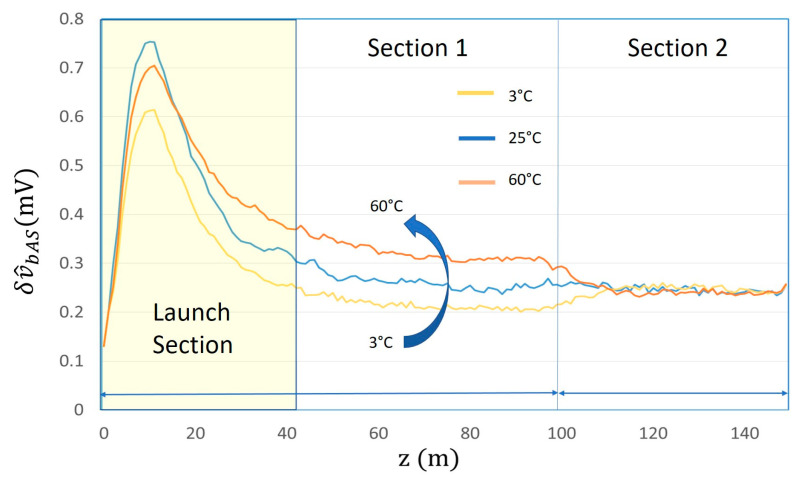
Behavior, as a function of z, and for different temperature values of fiber Section 1, of the difference ${\delta \hat{v}}_{bAS}$ between the values of the received Anti-Stokes backscattered signals correspondent to fiber sections $z_{i}$ and $z_{i-1}$ separated by a distance ∆z=1 m, with i=1,…N=150.

The result of such post-processing is shown in Figure 7, where the quantity ${\delta \hat{v}}_{bAS}$, proportional to $\delta {\hat{p}}_{bAS}$ and representing the Anti-Stokes signal as a function of z, is reported for different temperature values. In order to reduce noise, further averaging was performed with discretization of one meter.

The resolution of the system is defined by the pulse transactions, which in the case of the “Long Pulse” corresponds to the rise time. The rise time of 80 nsec generates a resolution of approximately 8 m, similar to that of the “Short Pulse”.

By comparing Figure 7 with Figure 3d, it is possible to appreciate the fact that the proposed “Long pulse” approach, which performs a subsequent differentiation of the received signal, also confirms from the experimental point of view its theoretically expected capability to detect the temperature variations along the fiber.

**Figure 8:**
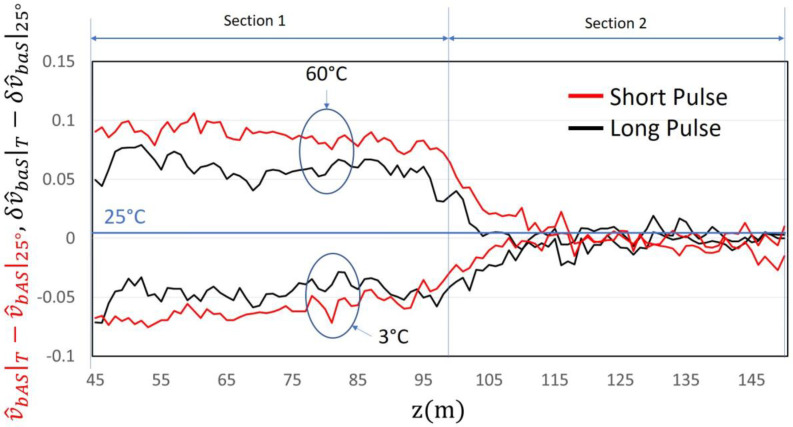
Comparison between experimental results in the “Short” and “Long pulse” operating procedures, normalized to 25 °C.

A further confirmation is represented by Figure 8, where a comparison of the experimental Anti-Stokes signals for “Short pulse” and “Long pulse” is presented. The correlation between temperature variation and measured signals is very similar in both cases, demonstrating the effectiveness of the new proposed method.

**Figure 9:**
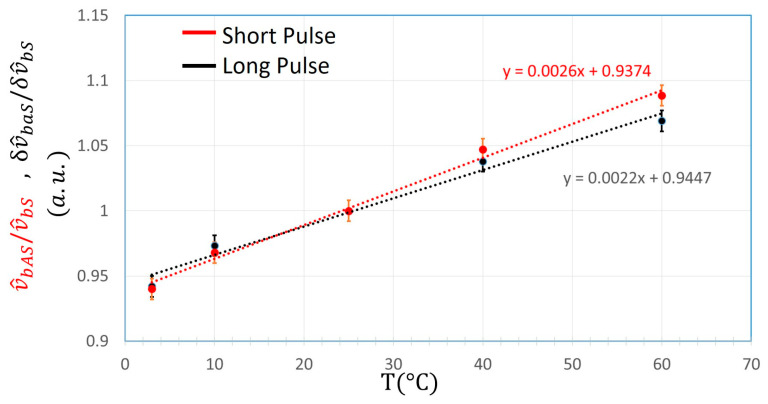
Temperature relationship of ${\hat{v}}_{bAS}$ normalized to ${\hat{\;v}}_{bS}$ for “Short pulse” (red line) and $\delta {\hat{v}}_{bAS}$ normalized to $\delta {\hat{\;v}}_{bS}$ for “Long pulse” (black line).

Once the use of the “Long pulse” was validated, further measurements were carried out to obtain the relationship between temperature and Anti-Stokes signal, as shown in Figure 9, for the “Short” and “Long Pulse” normalized to Stokes signal.

Each point was obtained by averaging the values of the optical fiber sections at the same temperature:

(18) $${\left({\hat{v}}_{baS}/{\hat{v}}_{bS}\right)}_{T}=\frac{\left(\sum_{i=J}^{i=k}{\left({\hat{v}}_{baS}/{\hat{v}}_{bS}\right)}_{T}{|}_{Z_{i}}\right)}{\left(k-j\right)}\;\mathrm{Short}\mathrm{Pulse}$$

(19) $${\left(\delta {\hat{v}}_{baS}/\delta {\hat{v}}_{bS}\right)}_{T}=\frac{\left(\sum_{i=J}^{i=k}{\left(\delta {\hat{v}}_{baS}/\delta {\hat{v}}_{bS}\right)}_{T}{|}_{Z_{i}}\right)}{\left(k-j\right)}\;\mathrm{Long}\mathrm{Pulse}$$

For fiber Section 1, the portion z∈[50 m,90 m] was considered, while for fiber Section 2 the stretch was z∈[110 m,150 m]. Data from fiber Section 2, which was set at 25 °C in every acquisition, were used to determine the standard error. Under these conditions, a standard error of ±3 °C was reached for “Long Pulse” and “Short Pulse”.

## 5. Conclusions

A preliminary realization of a Raman Distributed Temperature-Sensing System was demonstrated, tailored for the cases when the linear lengths of the regions to be monitored do not exceed a few hundred meters. Thanks to the accurate post-processing procedure that was proposed, pulses of space duration of the order of the fiber under test can be applied to the optical source, obtaining the same temperature characterizations as the cases when pulses of shorter duration and higher instantaneous power are applied. Furthermore, the resolution of the system is also the same, in our case approximately 8 m, and is linked only to the transaction phases of the optical pulses. Preliminary experimental measurements, validated by a rigorous simulation program, yield a sensitivity of 1.8 microvolts per degree of temperature variation.

The requirements on the electronic driving circuitry, which can be satisfied by typical commercial devices, combined with the exploitation of power lasers operating in the first optical window, allow the global expenditure related to the realization and operation of the proposed system to be maintained at low levels.

## Figures and Tables

**Table 1:** Laser pulse parameters.

Pulse	$t_{pulse}$ (ns)	$t_{rise}$ (ns)	$t_{fall}$ (ns)	$P_{IN}$ (W)	Period (ms)
Long	1550	80	42	1.70	1
Short	82	50	32	0.98	1

## Data Availability

The data presented in this study are available on request from the corresponding author.
